# Deficient Wnt Signaling and Synaptic Vulnerability in Alzheimer’s Disease: Emerging Roles for the LRP6 Receptor

**DOI:** 10.3389/fnsyn.2018.00038

**Published:** 2018-10-30

**Authors:** Johanna Buechler, Patricia C. Salinas

**Affiliations:** Department of Cell and Developmental Biology, University College London, London, United Kingdom

**Keywords:** synaptic degeneration, Alzheimer’s disease, amyloid-beta, Wnt signaling, LRP6

## Abstract

Synapse dysfunction and loss represent critical early events in the pathophysiology of Alzheimer’s disease (AD). While extensive research has elucidated the direct synaptotoxic effects of Amyloid-β (Aβ) oligomers, less is known about how signaling pathways at the synapse are affected by Aβ. A better understanding of the cellular and molecular mechanisms underlying synaptic vulnerability in AD is key to illuminating the determinants of AD susceptibility and will unveil novel therapeutic avenues. Canonical Wnt signaling through the Wnt co-receptor LRP6 has a critical role in maintaining the structural and functional integrity of synaptic connections in the adult brain. Accumulating evidence suggests that deficient Wnt signaling may contribute to AD pathology. In particular, LRP6 deficiency compromises synaptic function and stability, and contributes to Aß production and plaque formation. Here, we review the role of Wnt signaling for synaptic maintenance in the adult brain and the contribution of aberrant Wnt signaling to synaptic degeneration in AD. We place a focus on emerging evidence implicating the LRP6 receptor as an important modulator of AD risk and pathology.

## Introduction

Neurodegenerative diseases are characterized by progressive deterioration of nerve cell function and widespread neuronal death. Among them, Alzheimer’s disease (AD) represents the most common form of dementia, associated with debilitating mental and cognitive decline (Alzheimer’s Association, 2016). Due to changing demographics and increasing life expectancies, AD is rapidly rising in prevalence and its economic and social impact on our societies is predicted to soar in the coming decades (Hebert et al., 2001; Prince et al., 2013; Alzheimer’s Association, 2016). No disease-modifying treatments are available for AD. Therefore, there is an urgent need to develop effective therapeutic approaches to halt or slow disease progression. Intense research over the past decades has begun to reveal the complex mechanistic underpinnings of AD involving genetic risk, age-related cellular vulnerability and lifestyle factors. However, the molecular and cellular determinants of AD susceptibility or resilience and the key mechanisms that drive disease progression remain poorly understood.

AD presents a variety of pathological hallmarks including extracellular aggregations of amyloid-β (Aβ) protein, intracellular neurofibrillary tangles of tau protein and neuroinflammation mediated by glial cells. However, the best correlate of cognitive decline in AD patients is synapse loss, which occurs during early disease stages and precedes neuronal death (DeKosky and Scheff, 1990; Terry et al., 1991; Scheff et al., 1993; DeKosky et al., 1996; Selkoe, 2002). Synaptic impairments manifest early also in transgenic mouse models of AD (Knobloch and Mansuy, 2008; Hong et al., 2016a; Viana da Silva et al., 2016). Therefore, synaptic dysfunction and loss are key early events in the pathogenesis of AD. Protection of synapses and/or restoration of synaptic function is an attractive therapeutic strategy, as it may offer a chance of reversing or halting cognitive decline before the onset of large-scale and irreversible neuronal death. Understanding the molecular and cellular mechanisms that trigger synapse vulnerability in AD is critical for therapeutic development.

Research into the causes of synaptic degeneration in AD has focused on the role of soluble oligomers of Aβ protein. Aβ peptides, derived through enzymatic cleavage of the amyloid precursor protein (APP), self-aggregate into oligomers which are widely regarded as the principal pathogenic agents in AD (McLean et al., 1999; Näslund et al., 2000; Esparza et al., 2013). Indeed, Aβ oligomers exert a multitude of synaptotoxic effects (Palop and Mucke, 2010; Ferreira and Klein, 2011; Tu et al., 2014; Forner et al., 2017). Oligomeric Aβ accumulates at synaptic sites, impairs synaptic transmission and plasticity, compromises the structural integrity of dendritic spines and triggers spine and synapse elimination (Walsh et al., 2002; Hsieh et al., 2006; Shankar et al., 2008; Koffie et al., 2009; Wei et al., 2010; Pickett et al., 2016).

Several studies suggest that Aβ also initiates a variety of downstream events, which lead to the deregulation of key cellular pathways that control synaptic function and integrity. For example, Aβ downregulates the receptor tyrosine kinase EphB2, which is important for NMDA receptor trafficking to synaptic sites (Cissé et al., 2011; Miyamoto et al., 2016). Aβ also induces the aberrant re-activation of developmental mechanisms of synapse elimination, driven by complement proteins and microglia (Schafer and Stevens, 2010; Stephan et al., 2012; Hong et al., 2016b). Another downstream target of Aβ is the Wnt pathway, a key signaling cascade at the synapse. Growing evidence suggests that deficient Wnt signaling contributes to synaptic dysfunction and loss during AD pathogenesis. Multiple Aβ-targeting treatments have failed to produce appreciable cognitive benefits in AD clinical trials in recent years (Holmes et al., 2008; Doody et al., 2013, 2014; Vellas et al., 2013; Salloway et al., 2014). It is therefore critical to understand the downstream cellular responses to Aβ to identify new therapeutic targets. Here, we will summarize emerging evidence that deficient Wnt signaling could contribute to synaptic degeneration in AD, focusing on the key role of the Wnt co-receptor LRP6.

## Wnt Signaling and Synapse Health in the Adult Brain

Wnt proteins are a family of highly conserved secreted lipoproteins that activate several intracellular signaling pathways and control key aspects of development and tissue homeostasis in all metazoans (Gordon and Nusse, 2006; MacDonald et al., 2009; Nusse and Clevers, 2017). Wnt signaling plays a vital role in the nervous system, from embryogenesis to higher brain function in the adult.

**Figure 1:**
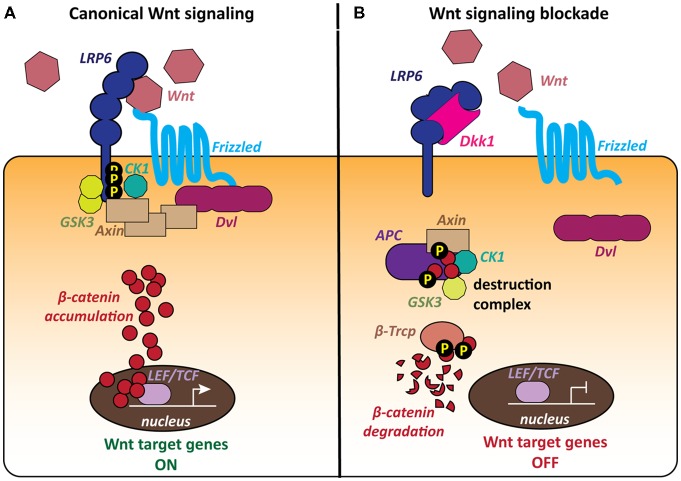
The canonical Wnt pathway and the role of the LRP6 receptor. The LRP6 receptor occupies a unique position within the canonical Wnt pathway as it is involved in both activation and blockade of the cascade. **(A)** The canonical Wnt cascade is activated by binding of secreted Wnt molecules to the Frizzled (Fz) receptor and LRP6 co-receptor at the cell surface. The formation of this complex leads to the recruitment of the scaffold protein Dishevelled (Dvl) and phosphorylation of the LRP6 C-tail by the kinases GSK3 and CK1. Cytoplasmic β-catenin accumulates and then translocates to the nucleus and activates Wnt target genes. **(B)** The secreted Wnt antagonist Dickkopf 1 (Dkk1) blocks Wnt signaling by binding to the LRP6 receptor, thereby preventing the formation of the Wnt-Fz-LRP6 complex. The cytoplasmic destruction complex containing Axin, APC, GSK3 and CK1 phosphorylates β-catenin, targeting it for proteasomal degradation. Wnt target genes remain repressed.

The most extensively studied signaling cascade activated by Wnts is the canonical or β-catenin pathway, which regulates the stability of the transcriptional co-activator β-catenin, thereby controlling the expression of Wnt target genes (Figure 1). Wnt ligands bind to Frizzled (Fz) receptors and LRP5/6 co-receptors resulting in the formation of a ternary complex of Wnt, Fz and LRP5/6 at the cell surface (MacDonald et al., 2009; Clevers and Nusse, 2012; MacDonald and He, 2012; Nusse and Clevers, 2017). The downstream signaling cascade from this receptor complex leads to recruitment of the scaffold protein Dishevelled (Dvl) to the plasma membrane and inhibition of GSK3β. β-catenin can then accumulate in the cytoplasm and translocate to the nucleus, where it regulates the expression of Wnt target genes in association with TCF/LEF transcription factors (Figure 1A; Cong et al., 2003; Valenta et al., 2012; Schuijers et al., 2014). When the pathway is inactive, cytosolic β-catenin is degraded by a macromolecular destruction complex composed of the scaffold protein Axin, the tumor suppressor protein APC and the two constitutively active serine-threonine kinases CK1 and GSK3ß (Figure 1B).

The Wnt signaling pathway is regulated by a range of secreted endogenous antagonists, that either modify or sequester Wnt ligands or modulate Wnt-receptor interactions at the plasma membrane (Cruciat and Niehrs, 2013). The best characterized Wnt antagonists are the Dickkopf (Dkk) proteins, a small evolutionarily conserved family of secreted glycoproteins with four members in vertebrates, Dkk1–4, (Niehrs, 2006; Cruciat and Niehrs, 2013). Dkk1 in particular is well characterized as a major endogenous Wnt antagonist. Dkk1 inhibits Wnt signaling by binding to the Wnt co-receptor LRP6, preventing the formation of the Wnt-Fz-LRP6 complex and the activation of the Wnt cascade (Figure 1B; Bafico et al., 2001; Mao et al., 2001; Semënov et al., 2001). Thus, increased levels of Dkk1 would decrease the availability of the LRP6 receptor for Wnt ligands.

During postnatal brain development, Wnts play an important role for synapse assembly in the CNS (reviewed in Ille and Sommer, 2005; Salinas and Zou, 2008; Budnik and Salinas, 2011; Park and Shen, 2012; Salinas, 2012; Stamatakou and Salinas, 2014). Many Wnt signaling components are also expressed in the adult mammalian nervous system (Shimogori et al., 2004; Wayman et al., 2006; De Ferrari et al., 2007; Gogolla et al., 2009; Sahores et al., 2010; Seib et al., 2013; Zhang et al., 2014). Accumulating evidence indicates a significant contribution of Wnt signaling to synapse physiology and stability in the adult brain.

Wnt signaling is important for synaptic transmission at mature synapses. Blockade of endogenous Wnts through sFRP Wnt antagonists, which sequester Wnt ligands in the extracellular space, reduces glutamatergic neurotransmission in cultured neurons (Varela-Nallar et al., 2010; Cerpa et al., 2011; Ciani et al., 2015). In contrast, application of exogenous Wnts acutely enhances excitatory synaptic transmission in mature hippocampal neurons (Beaumont et al., 2007; Cerpa et al., 2008; Avila et al., 2010; Varela-Nallar et al., 2010; Ciani et al., 2011, 2015). *In vivo*, genetic deficiency in *Wnt7a* and *Dvl1* results in reduced synaptic vesicle numbers and decreased transmitter release (Ahmad-Annuar et al., 2006; Ciani et al., 2015). Therefore, Wnts are required in mature neuronal circuits for synaptic function.

Wnt signaling also contributes to the structural stability of established synaptic connections (reviewed in Dickins and Salinas, 2013). Short-term blockade of canonical Wnt signaling through the secreted Wnt antagonist Dkk1 induces the disassembly of excitatory synapses in mature hippocampal neurons (Purro et al., 2012). Dkk1 decreases the number and size of pre- and postsynaptic protein clusters through the rapid dispersal of synaptic components, without affecting cell viability (Purro et al., 2012). Electron microscopy analyses show that remaining synapses have smaller active zones and postsynaptic densities, suggesting that Wnt blockade induces coordinated shrinkage and elimination of pre- and postsynaptic sites (Purro et al., 2012). *In vivo* suppression of Wnt signaling through inducible expression of Dkk1 in the adult brain of transgenic mice recapitulates these effects by eliciting synapse degeneration (Galli et al., 2014; Marzo et al., 2016). In the striatum, Wnt inhibition by Dkk1 leads to the loss of excitatory cortico-striatal as well as dopaminergic synapses and reduced neurotransmission at remaining synapses (Galli et al., 2014). In the hippocampus, Dkk1 triggers excitatory synapse loss, accompanied by defects in synaptic plasticity (blocked LTP and enhanced LTD) and memory function (Marzo et al., 2016). Synaptic degeneration occurs in the absence of cell death, indicating that Dkk1-mediated Wnt blockade directly compromises synapse stability and integrity (Galli et al., 2014; Marzo et al., 2016). Collectively, these findings demonstrate that endogenous Wnt signaling is required for synapse stability in the mature brain.

The physiological relevance of Wnt-regulated synaptic integrity is underscored by the implication of Wnt signaling in memory formation (reviewed in Oliva et al., 2013). Several studies have shown that blockade of Wnt signaling in the adult brain interferes with memory function. During fear memory consolidation in the amygdala, a transient increase in both β-catenin and Wnt target gene expression is observed (Maguschak and Ressler, 2011). In contrast, local injection of the Wnt antagonist Dkk1 disrupts memory consolidation (Maguschak and Ressler, 2011). A separate study similarly showed that administration of Wnt3a antibody or Wnt antagonists sFRP1 or Dkk1 block fear memory acquisition and consolidation, whereas infusion of exogenous Wnt3a enhances memory formation (Xu et al., 2015). Post-training infusion of Dkk1 also interferes with memory consolidation in a hippocampus-dependent object recognition task (Fortress et al., 2013). *In vivo* induction of Dkk1 expression in the adult hippocampus leads to memory deficits in a range of paradigms, including spatial navigation and fear memory (Marzo et al., 2016). Together, these studies show that Wnt signaling is required for synaptic changes underlying memory formation and consolidation.

## Aberrant Wnt Signaling Contributes to Synaptic Vulnerability in AD

Accumulating evidence suggests that aberrant Wnt signaling plays a role in the pathogenesis of AD (reviewed in De Ferrari et al., 2014; Inestrosa and Varela-Nallar, 2014; Purro et al., 2014; Libro et al., 2016; García-Velázquez and Arias, 2017). Multiple findings indicate that Wnt signaling becomes deregulated in the context of AD. In brains of familial AD patients, active GSK3β accumulates, whereas β-catenin levels are reduced (Zhang et al., 1998; Pei et al., 1999; Kawamura et al., 2001). Dkk1 expression is increased in brains from AD patients and transgenic AD mouse models (Caricasole et al., 2004; Rosi et al., 2010). Dkk3, which is closely related to Dkk1, is also elevated in plasma and cerebrospinal fluid of AD patients (Zenzmaier et al., 2009). Moreover, exposure of hippocampal neurons to Aβ leads to inhibition of Wnt signaling (De Ferrari et al., 2003; Alvarez et al., 2004). Consistent with these findings, Aß rapidly increases the levels of the endogenous Wnt antagonist Dkk1 (Purro et al., 2012). Together, these results indicate a deficiency of Wnt signaling in the context of AD.

How does perturbation of Wnt signaling influence AD pathogenesis? Suppression of Wnt signaling by Aß could decrease Wnt-dependent synaptic stability during the early stages of AD. Consistent with this hypothesis, short-term exposure of hippocampal neurons to oligomeric Aβ induces Dkk1 expression and triggers synapse loss (Purro et al., 2012; Sellers et al., 2018). Importantly, neutralizing antibodies against Dkk1 protect synapses from Aβ toxicity (Purro et al., 2012). Interestingly, a recent study suggests that Dkk1 may signal through its receptor Kremen1 to mediate Aβ synaptotoxicity (Ross et al., 2018). In summary, these findings strongly suggest that Dkk1 is involved in Aß-mediated synapse loss.

To mimic the effect of Aß *in vivo*, a transgenic mouse model that inducibly expresses Dkk1 was generated. Inhibition of endogenous Wnt signaling by Dkk1 *in vivo* triggers synapse loss in the striatum and hippocampus (Galli et al., 2014; Marzo et al., 2016). Hippocampal Dkk1 expression reduces synaptic transmission, impairs LTP and enhances LTD, and these synaptic defects are accompanied by memory deficits (Marzo et al., 2016). Thus, inducible Dkk1 expression in the adult brain mimics various aspects of Aβ-induced synaptic degeneration. These findings support the view that suppression of Wnt signaling downstream of Aβ promotes synaptic destabilization and may contribute to cognitive decline.

## The LRP6 Receptor as a Key Emerging Link Between Wnt Signaling, Synapse Health and AD

Activation of the canonical Wnt pathway requires the cooperation of Wnt/Fz with the single-pass transmembrane co-receptors LRP5/6, which belong to the extended low-density lipoprotein receptor (LDLR) family (He et al., 2004; MacDonald and He, 2012). LRP5 and LRP6 are highly homologous and display largely overlapping expression patterns, however LRP6 plays a more influential role and is the best characterized Wnt co-receptor (MacDonald et al., 2011). LRP6 is key for Wnt signal transduction, which relies on the phosphorylation of the LRP6 intracellular domain following Wnt binding to this co-receptor (He et al., 2004; MacDonald and He, 2012). LRP6 is also the main receptor for the Dkk family of Wnt secreted antagonists, most notably Dkk1 (Bafico et al., 2001; Mao et al., 2001; Semënov et al., 2001). Dkk1 disrupts LRP6 function as a Wnt co-receptor. The LRP6 receptor therefore occupies a unique position within the Wnt pathway (Figure 1).

### The Role of LRP6 in Embryogenesis and Synapse Formation

Constitutive knockout of LRP6 is lethal, as *LRP6^−/–^* mice die at birth due to early patterning defects (Pinson et al., 2000). Loss of function of LRP6 causes severe defects specifically in the nervous system, including reduced production of granule cells in the hippocampus, deficiency in thalamic development and impaired neuronal proliferation in the neocortex (Zhou et al., 2004a,b, 2006). Therefore, LRP6 plays a critical role during early development, consistent with its role as a key Wnt receptor in the canonical Wnt pathway.

LRP6 is also important during synapse formation in the developing nervous system. Knockdown of LRP6 in young hippocampal neurons leads to a decrease in the number of excitatory synapses and impairs spine morphogenesis, without affecting inhibitory synapses (Sharma et al., 2013). Similarly, *in vivo* loss of function of LRP6 in developing cortical neurons compromises dendritic spine development, accompanied by a functional impairment of excitatory postsynaptic currents (Sharma et al., 2013). LRP6 phosphorylation at residue S1490, which is a key step during Wnt signal transduction, is required for LRP6-mediated synaptogenesis (Sharma et al., 2013). Thus, LRP6 has a key role in Wnt-mediated synapse formation and is required for excitatory synapse formation during postnatal development.

### LRP6 and Synapse Integrity in the Adult and Aging Brain

**Figure 2:**
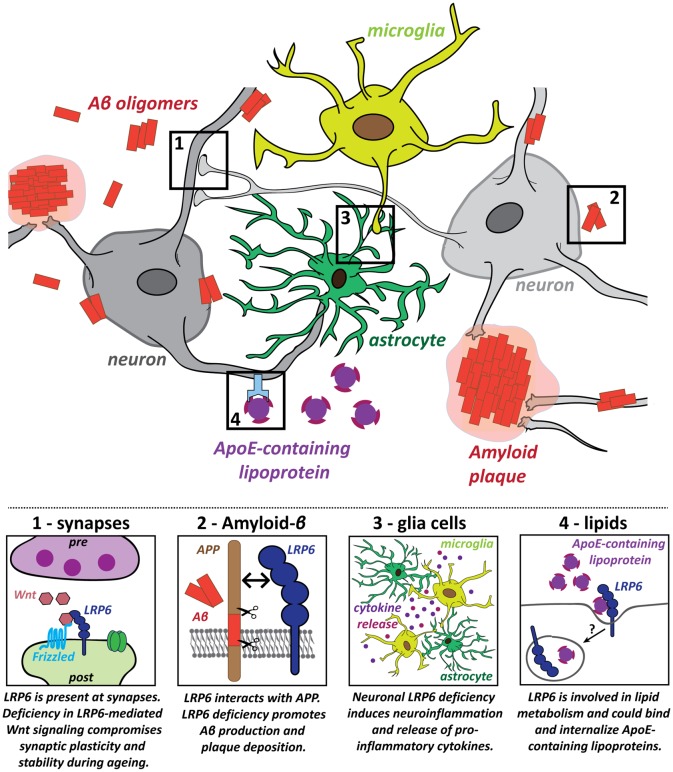
Potential roles for the LRP6 receptor in several key pathogenic processes in Alzheimer’s disease (AD). Several hallmarks of AD pathology have been identified and LRP6 dysfunction contributes to these pathogenic processes in multiple different ways: (1) Deficiency in LRP6-mediated Wnt signaling increases synaptic vulnerability, which could promote synaptic dysfunction and loss in AD; (2) LRP6 regulates amyloid precursor protein (APP) processing and LRP6 loss of function increases amyloid-β (Aβ) generation and plaque formation; (3) Neuroinflammatory processes also contribute to AD pathology. Neuronal LRP6 deficiency has a secondary impact on microglia and astrocytes, promoting neuroinflammation and secretion of pro-inflammatory molecules; (4) Lipid metabolism is deregulated in AD and *Apolipoprotein E* (*ApoE*) is a major genetic risk factor for AD. LRP6 could participate in cholesterol transport and may be involved in the internalization of ApoE-containing lipoprotein particles.

The role of LRP6 in the mature brain was investigated in a conditional knockout (cKO) model, based on CaMKII-Cre-driven deletion of LRP6 in principal forebrain neurons (Liu et al., 2014). LRP6 cKO impairs Wnt signaling and causes synapse dysfunction and loss in an age-dependent manner. Spine density, synaptic protein levels, synaptic plasticity and memory function are normal in young adult cKO mice at 6 months (Liu et al., 2014). However, LRP6 cKO mice at 18–22 months exhibit spine loss in hippocampal and cortical neurons, impaired LTP maintenance and memory deficits in fear conditioning tests (Liu et al., 2014). Reduced cell viability may contribute to these effects (Liu et al., 2014). Thus, neuronal LRP6 deficiency leads to synaptic dysfunction and loss, accompanied by cognitive impairment, specifically in the aging brain (Figure 2, panel 1).

### LRP6 and Links to AD

Several studies suggest a link between LRP6 and AD. Through genome-wide linkage analyses, a broad susceptibility locus for late-onset AD was identified on chromosome 12, which includes the region encoding *LRP6* (Pericak-Vance et al., 1997; Rogaeva et al., 1998; Kehoe et al., 1999; Scott et al., 2000; Mayeux et al., 2002; Myers et al., 2002; D’Introno et al., 2006; Lee et al., 2008). Follow-up studies associated two LRP6 single-nucleotide polymorphisms (SNPs) and one alternative splice variant of LRP6 with an increased risk of late-onset AD (De Ferrari et al., 2007; Alarcón et al., 2013). These observations establish a genetic link between late-onset AD and LRP6. Both the LRP6 14e-Val variant and the LRP6Δ3 splice isoform, which skips exon 3, are associated with reduced activation of Wnt/β-catenin signaling in HEK293T cells (De Ferrari et al., 2007; Alarcón et al., 2013).

In human AD brains, LRP6 mRNA and protein levels are significantly downregulated compared to age-matched controls (Liu et al., 2014). Concomitant reduction in canonical Wnt signaling suggests that LRP6-mediated Wnt signaling is compromised in AD (Liu et al., 2014). LRP6 deficiency promotes AD pathology as the levels of soluble Aβ_40/42_ and amyloid plaque burden are increased in hippocampus and cortex of *APP/PSEN1* transgenic mice lacking neuronal LRP6 (Figure 2, panel 2; Liu et al., 2014). LRP6 cKO also exacerbates memory deficits in this AD model (Liu et al., 2014). Together, these findings indicate that LRP6 deficiency—as observed in AD brains—aggravates AD pathology.

How does LRP6 modulate amyloid pathology? LRP6 can directly influence the processing of APP (Figure 2, panel 2). Co-immunoprecipitation assays demonstrated that APP and LRP6 interact via their extracellular domains (Liu et al., 2014). Knockdown of LRP6 in an APP-expressing cell line increases production of Aβ and its by-products, suggesting that LRP6 downregulation stimulates amyloidogenic processing of APP. *In vivo* LRP6 deficiency similarly increases endogenous Aβ_40_ and Aβ_42_ levels, whereas overexpression of LRP6 leads to reduced production of Aβ_40_ and Aβ_42_ (Figure 2, panel 2, Liu et al., 2014). LRP6 may modulate Aβ production by promoting cell surface localization of APP (Liu et al., 2014), which favors non-amyloidogenic processing by α-secretase (Haass et al., 1993; Parvathy et al., 1999; Carey et al., 2005; Zhang and Song, 2013). These results suggest that LRP6 interacts with APP to retain it at the cell surface, thereby suppressing Aβ production. In contrast, LRP6 deficiency in AD models increases the amyloidogenic processing of APP, fueling Aβ generation and plaque formation. Therefore, LRP6 loss of function could be part of a positive feedback loop leading to the exacerbation of AD pathology.

LRP6 may also play a role in other AD-relevant processes, beyond synaptic maintenance and Aβ pathology. First, the influence of non-neuronal glia cells and aberrant immune responses are increasingly recognized as important components of AD pathology (Heneka et al., 2015; Heppner et al., 2015; Zhang and Jiang, 2015; Bronzuoli et al., 2016; De Strooper and Karran, 2016). Genetic, clinical and cell biology data have shown that neuroinflammation driven by microglia and astrocytes plays a significant causal role in driving and exacerbating AD pathogenesis (Heneka et al., 2015; Heppner et al., 2015; Zhang and Jiang, 2015; Bronzuoli et al., 2016). Mutations in several microglia-associated genes are linked to late-onset AD, indicating a central role for microglia in the disease etiology (Jones et al., 2010; Malik et al., 2015; Villegas-Llerena et al., 2016; Efthymiou and Goate, 2017). In addition, microglia may contribute to synapse elimination downstream of Aβ (Paolicelli et al., 2011; Hong et al., 2016a,b). Intriguingly, postnatal neuronal deletion of LRP6 in conditional KO mice increases the presence of astrocytes and microglia in the aging hippocampus and results in the expression of pro-inflammatory cytokines (Figure 2, panel 3; Liu et al., 2014). These findings suggest that deficiency in neuronal LRP6-mediated Wnt signaling could indirectly promote neuroinflammation during aging, thereby further fueling AD pathogenesis. LRP6 is also expressed in microglia and astrocytes (Halleskog et al., 2011; L’Episcopo et al., 2011; Zhang et al., 2014; Zeisel et al., 2015). However, the role of LRP6 in glia cells in the context of AD remains to be studied. Given that astrocytes have important functions in regulating synaptic transmission and plasticity, they could also contribute to the synaptic defects observed in early stages of AD. Future studies on the impact of LRP6-mediated Wnt signaling on astrocytes could therefore also help to elucidate changes in the astrocyte-neuron crosstalk at synapses in the context of AD.

LRP6 deficiency could further contribute to AD pathogenesis through its role in lipid metabolism (Figure 2, panel 4). As a member of the extended LDLR receptor family, LRP6 is involved in LDL uptake and cholesterol clearance (Liu et al., 2008; Tomaszewski et al., 2009; Go and Mani, 2012; Ye et al., 2012). Coding-region LRP6 SNP mutations have been associated with elevated LDL cholesterol levels (Mani et al., 2007; Liu et al., 2008; Tomaszewski et al., 2009; Go et al., 2014). Perturbation of lipid metabolism is associated with AD, as gene network analyses have highlighted altered lipid and cholesterol metabolism as one of three biological pathways with a central role in AD etiology (Jones et al., 2010; Guerreiro et al., 2013; Guerreiro and Hardy, 2014; Hardy et al., 2014; International Genomics of Alzheimer’s Disease Consortium (IGAP), 2015; Karch and Goate, 2015; Efthymiou and Goate, 2017). High cholesterol levels in mid-life may contribute to AD susceptibility (Notkola et al., 1998; Kivipelto et al., 2002; Whitmer et al., 2005). The primary function of the major AD risk gene Apolipoprotein E (*APOE*) is in cholesterol transport (Huang and Mahley, 2014), and ApoE-containing lipoproteins bind to all core members of the LDLR receptor family, to LRP5 and likely to LRP6 as well (Figure 2, panel 4; Kim et al., 1998; Magoori et al., 2003; Beffert et al., 2004; Andersen and Willnow, 2006; Jaeger and Pietrzik, 2008; Guttman et al., 2010). These observations raise the intriguing possibility that LRP6 may also be involved in AD pathogenesis through deregulation of lipid metabolism. Altogether, these findings highlight the importance of LRP6 function and LRP6-mediated Wnt signaling for a healthy aging brain.

## Concluding Remarks

Long-term synaptic integrity and resilience are vital factors determining the susceptibility to an age-related degenerative disorder such as AD. Indeed, synaptic degeneration is a key early event in the pathophysiology of AD, and exciting new research is shedding more light on important cellular and molecular mechanisms that underpin synaptic vulnerability. Wnt signaling and the Wnt co-receptor LRP6 have a central role in synapse formation, stability and function. Growing evidence indicates that deficiency of Wnt signaling contributes to synapse dysfunction and loss in the adult and aging brain, fueling the pathogenesis of AD. Increased levels of the Wnt antagonist Dkk1 or LRP6 loss of function negatively impact on several key pathogenic processes by inducing synapse dysfunction and degeneration as well as deficits in LTP and memory. LRP6 deficiency also promotes Aβ production and amyloid deposition, stimulates neuroinflammation and potentially contributes to aberrant lipid metabolism. These findings point to Dkk1 and LRP6 as important molecular modulators of AD risk and pathology. Restoring Wnt signaling and LRP6 function may therefore represent a viable therapeutic strategy for AD.

## Author Contributions

JB and PS designed the outline of the article, wrote the manuscript and revised the article. JB created the figures.

## Conflict of Interest Statement

The authors declare that the research was conducted in the absence of any commercial or financial relationships that could be construed as a potential conflict of interest.
